# Supplementary material for: Global hypo-methylation in a proportion of glioblastoma enriched for an astrocytic signature is associated with increased invasion and altered immune landscape
Source: eLife. 2022 Nov 22;11:e77335. doi: 10.7554/eLife.77335 (PMC9681209; doi:10.7554/eLife.77335)
Supplement: Figure 2—source data 1. [file elife-77335-fig2-data1.zip › Figure_2_source_data_1/Figure_2A/homerResults/motif23.similar.html]

motif23

## Information for motif23

C
T
G
A
C
T
G
A
T
G
A
C
C
T
G
A
T
C
A
G
C
T
G
A
T
A
G
C
T
C
A
G
T
C
G
A
T
C
G
A
T
C
G
A
C
T
A
G
A
C
T
G
A
C
G
T
A
T
G
C
  
Reverse Opposite:  

T
A
C
G
T
G
C
A
T
G
A
C
G
A
T
C
A
G
C
T
A
G
C
T
A
G
C
T
A
G
T
C
A
T
C
G
G
A
C
T
A
G
T
C
G
A
C
T
A
C
T
G
G
A
C
T
A
G
C
T
  

|  |  |
| --- | --- |
| p-value: | 1e-19 |
| log p-value: | -4.478e+01 |
| Information Content per bp: | 1.592 |
| Number of Target Sequences with motif | 33.0 |
| Percentage of Target Sequences with motif | 1.42% |
| Number of Background Sequences with motif | 6.3 |
| Percentage of Background Sequences with motif | 0.17% |
| Average Position of motif in Targets | 109.0 +/- 50.7bp |
| Average Position of motif in Background | 111.1 +/- 77.9bp |
| Strand Bias (log2 ratio + to - strand density) | -0.1 |
| Multiplicity (# of sites on avg that occur together) | 1.00 |
| Motif File: | file (matrix) reverse opposite |

### Similar de novo motifs found

|  |  |  |  |  |  |  |  |
| --- | --- | --- | --- | --- | --- | --- | --- |
| Rank | Match Score | Redundant Motif | P-value | log P-value | % of Targets | % of Background | Motif file |
| 1 | 0.620 | C G T A A T G C G T A C G A C T G T C A A C G T A G T C A G T C A C G T A G T C | 1e-12 | -28.081393 | 0.69% | 0.06% | motif file (matrix) |
